# Oral pre-administration of Purslane polysaccharides enhance immune responses to inactivated foot-and-mouth disease vaccine in mice

**DOI:** 10.1186/s12917-019-1782-3

**Published:** 2019-01-25

**Authors:** Rui Zhao, Xiangyu Meng, Guiyan Jia, Yongzhong Yu, Bocui Song

**Affiliations:** 1 Department of Pharmaceutical Engineering, College of Life Science & Biotechnology, Heilongjiang August First Land Reclamation University, Daqing High-Tech Industrial Development Zone, Daqing, 163319 People’s Republic of China; 2 Department of Gynaecology and Obstetrics, Daqing Oilfield Hospital, Daqing, 163311 People’s Republic of China

**Keywords:** Purslane polysaccharide, Foot-and-mouth disease vaccine, Immune, Immunostimulator

## Abstract

**Background:**

Foot-and-mouth disease (FMD) is one of the greatest disease threats to animal husbandry worldwide. Though various vaccines against foot-and-mouth disease virus (FMDV) have been developed, vaccine effectiveness is still not satisfactory. In this work, we studied the potential ability of Purslane polysaccharide (POL-P3b) as a nutrient food additive to enhance immune responses to FMD vaccination in mice.

**Results:**

Our results demonstrated that oral administration of POL-P3b at mid- and high-doses significantly enhanced the FMDV-specific cellular and humoral immune responses in mice and increased the concentration of Ca^2+^ in lymphocytes. Importantly, POL-P3b could promote intestinal DC maturation and stimulate the secretion of intestinal SIgA in a dose-dependent manner. Moreover, the acute toxicity study showed that POL-P3b was non-toxic and safe in mice.

**Conclusion:**

Our findings provided solid evidence that POL-P3b might be a novel immunostimulator and a boosting agent for increasing the efficacy of FMD vaccination, and the mechanism was related to stimulating the intestinal mucosal immune function that subsequently enhanced the efficacy of FMD vaccination through pre-administration of oral POL-P3b.

## Background

It is well known that the immune system plays an important role in resisting the invasion of pathogenic microorganisms to maintain animal health in animal husbandry. Good nutrition is necessary for animals to maintain normal immune system function to inhibit the invasion of pathogenic bacteria or viruses. In recent years, the animal epidemic situation is becoming more severe and complex due to frequent outbreaks of various animal diseases, causing great loss to animal industry. Decline in immune function in animals has been recognized to be a key reason for animal disease outbreaks. Therefore, in recent years, researchers are increasingly paying attention to optimizing food additives in order to improve immune function for preventing and treating diseases in animal husbandry.

Foot-and-mouth disease (FMD) is a common infectious disease of cloven-hoofed animal caused by the foot-and-mouth disease virus (FMDV), which is listed as a notifiable animal disease by the World Organization for Animal Health (OIE). At present, both the O and A type of FMD are commonly found in China and pose a great threat to the development of animal husbandry through various routes of transmission. From 2005 to 2017, there were 125 outbreaks of FMD in China [1]. The development and use of inactivated vaccines against FMDV is the feasible way to control the outbreaks. FMDV interaction with lymphocytes and dendritic cells (DCs) in swine and cattle has been previously reviewed [2–5]. The intestinal tract is not only a place for digestion, absorption of nutrients, but also has an important immune function, which is the body’s first defendant line against infection. DCs are also important components of the intestinal immune system. The interaction between DCs and various cytokines in the surrounding environment affects the immune response and immune tolerance of the intestinal tract [6].

To induce immune responses effectively, the addition of nutrients has been considered as an important strategy to improve vaccine efficacy against FMDV infection [7]. It is proven that Chinese herbal medicine can be used as nutrient additives with some advantages, such as abundant natural resources, reliable efficacy and lower side effects [8, 9].

Purslane (*Portulaca oleracea*) an annual, succulent plant is known as a longevity food with high nutritional value and is a widespread medicinal herb that has been used as an edible plant and in traditional medicine to remedy a wide spectrum of diseases in different countries [10, 11]. In previous studies, we found that Purslane polysaccharide (POL-P3b), an active ingredient isolated from Purslane, possessed a wide range of bioactivities, such as enhancement of antitumor immunity and toxin reduction in vivo [12, 13]. In this study, we further attempted to evaluate the effects of pre-administration of oral POL-P3b as an immunostimulator on FMD vaccines. This will provide more evidence for the application of POL-P3b in animal husbandry.

## Results

### Immunoprotection against FMDV in challenged mice

**Fig. 1:**
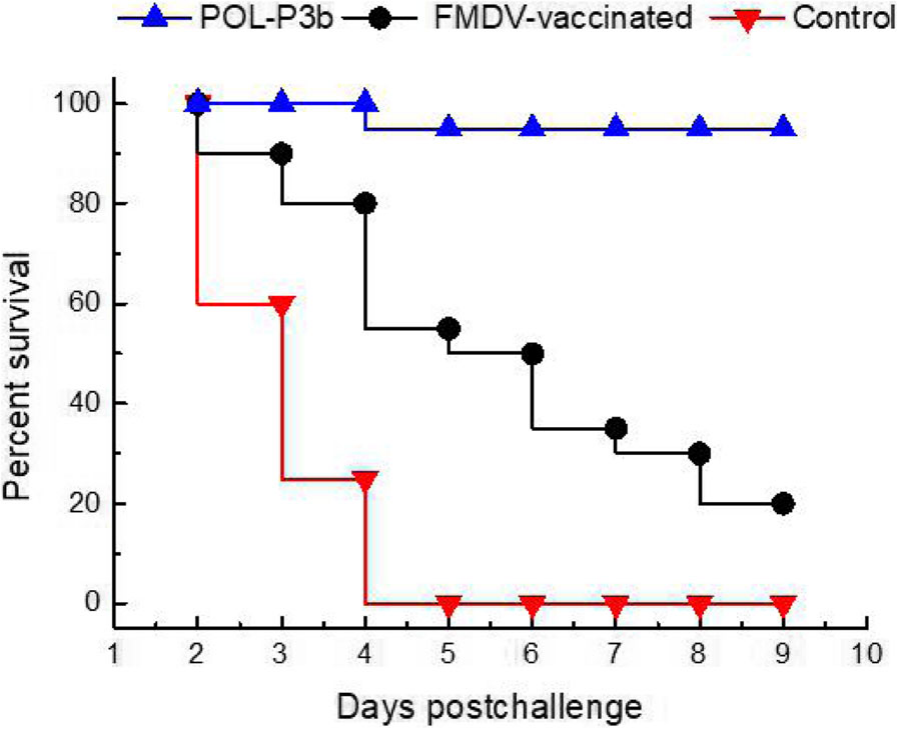
Protection against FMDV in challenged mice. Mice (*n* = 20) were immunized twice with FMDV, and then challenged 21 days post first immunization with 10^5.0^ TCID_50_/ 0.1 mL infectious FMDV O1C serotype by the i.p. route

To compare the protection ability after vaccination, the survival rate was observed following a vaccination and viral challenge of mice (21 days after vaccination). As showed in Fig. 1, the survival rate was highest in POL-P3b group (95%), and only 20% of mice in the FMDV-vaccinated group were protected. In contrast, all mice in control group (without FMD vaccination and POL-P3b--pretreated) were dead at 4 days post challenge.

### Effects of POL-P3b on IgG and isotypes in mice immunized with O-type FMD inactivated vaccine

**Fig. 2:**
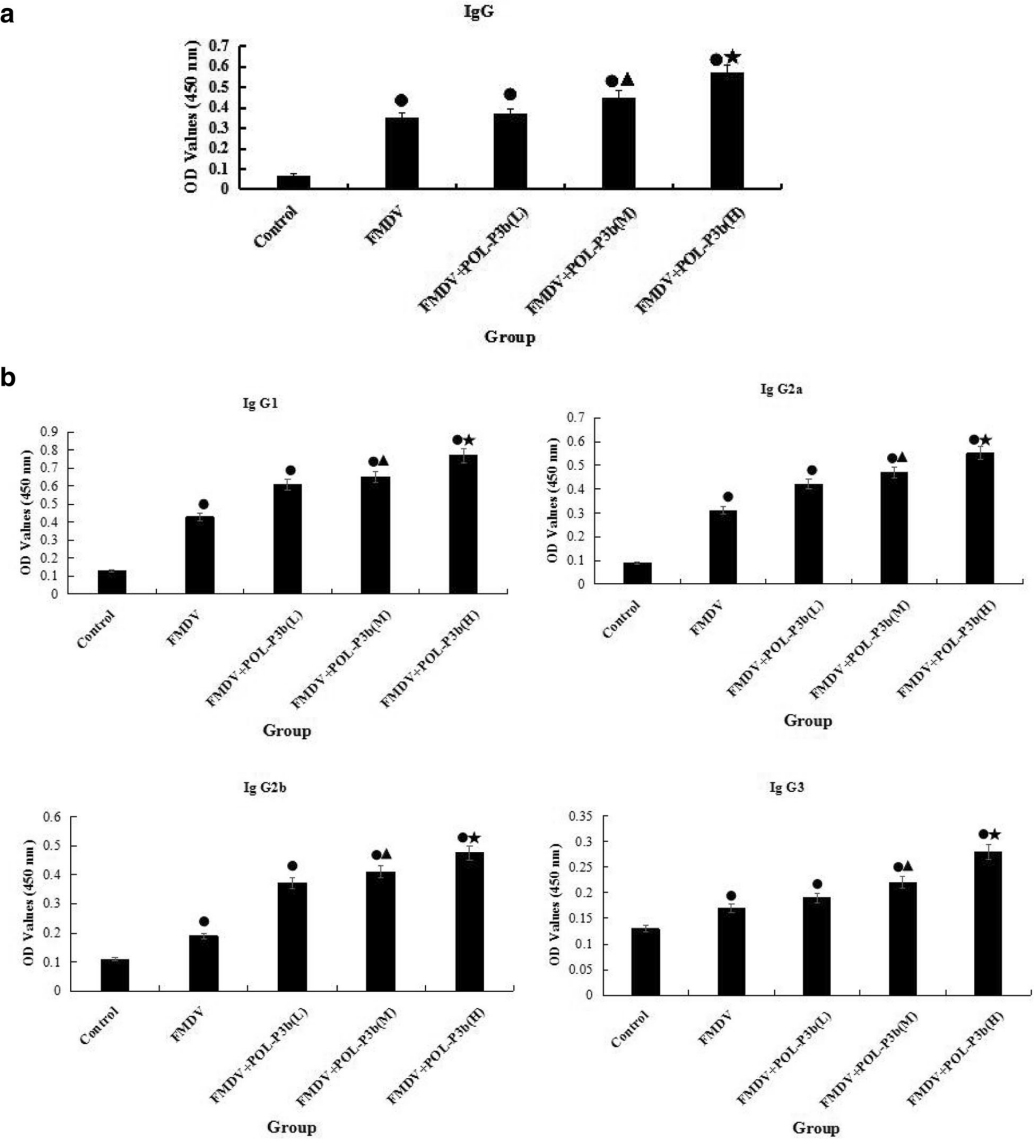
Effects of POL-P3b on IgG and isotype in mice immunized with O type FMD inactivated vaccine. **a** Effects of POL-P3b on IgG. **b** Effects of POL-P3b on IgG isotypes. Data were expressed as mean ± SD (*n* = 10). ^●^*P* < 0.01, compared with control; ^▲^*P* < 0.05, ^★^*P* < 0.01, compared with FMDV

Induction of humoral immunity is often used to evaluate the efficacy of FMD vaccines. The mice were immunized with FMD vaccines after POL-P3b administration. Antigen-specific serum antibodies were measured 2 weeks following booster vaccination via ELISA. Compared to mice in control group, a significant IgG response was found in mice immunized with FMD vaccines (Fig. 1). Mice treated with low-dose POL-P3b did not show difference in the level of total IgG relative to mice in FMDV group (Fig. 2a). However, mice treated with medium- and high-dose POL-P3b did show significantly higher levels of total IgG compared to mice in FMDV group (*P* < 0.05) (Fig. 2a). Furthermore, higher levels of all four IgG subclasses (IgG1, IgG2a、IgG2b and IgG3) were found in mice treated with medium- and high-dose POL-P3b (M, H) in contrast to mice in FMDV group (*P* < 0.05) (Fig. 2b). The results suggest that POL-P3b could stimulate the FMDV-specific antibody production in mice immunized with FMD vaccines.

### Effects of POL-P3b on lymphocyte proliferation and subpopulations in mice immunized with O-type FMD inactivated vaccine

The splenocyte proliferation is directly proportional to the strength of the immune response. The effect of POL-P3b on splenocyte proliferation in response to ConA and LPS stimulus was listed in Table 1. It was found that the proliferation of B cells in mice immunized with FMD vaccines increased significantly compared to control group (*P* < 0.05), and pre-administration of POL-P3b could remarkably enhance the proliferation of B cells in mice (Table 1). Interestingly, FMD immunization alone did not induce the proliferation of T cells, but pre-administration of medium- and high-dose POL-P3b could significantly induce the proliferation of T cells in mice (*P* < 0.05, *P* < 0.01) (Table 1). After co-stimulation of FMD vaccines with POL-P3b, the proliferation of B cells and T cells in mice were both higher than that in FMDV group (*P* < 0.05, *P* < 0.01). The results indicate that oral administration with POL-P3b could enhance immune response in mice immunized with FMD vaccines.

**Table 1:** Effect of POL - P3b on T cell and B cell proliferation in mice ($\overline{\mathrm{x}}\pm \mathrm{s}$)

Group	T cells	B cells
Control	1.03 ± 0.03	1.14 ± 0.21
FMDV	1.04 ± 0.02	1.52 ± 0.15*
FMDV+POL-P3b (L)	1.07 ± 0.04	1.54 ± 0.12**
FMDV+POL-P3b (M)	1.10 ± 0.03*^∆^	1.75 ± 0.16*^∆^
FMDV+POL-P3b (H)	1.35 ± 0.05**^∆∆^	1.97 ± 0.17**^∆^

Compared with control, *P < 0.05,**P < 0.01;Compared with FMDV, ^∆^*P* < 0.05, ^∆∆^*P* < 0.01. Data are presented as mean ± SD (*n* = 10)

T cell-mediated immunity plays a pivotal role for removing invading pathogen, and mature T cells are divided into CD4^+^ T cells and CD8^+^ T cells subgroups. We observed which type of T cells, CD4^+^ T cells or CD8^+^ T cells, played a critical role in immune response in mice immunized with the FMD vaccines, and the results were listed in Table 2. It was found that the percentage of CD4^+^ T cells increased significantly in mice immunized with FMD vaccines compared to control (*P* < 0.05), but no change was found in the percentage of CD8^+^ T cells and CD4^+^/CD8^+^ ratio. In addition, the percentage of CD4^+^ T and CD8^+^ T cells significantly increased in immunized mice pre-treated with high-dose POL-P3b (*P* < 0.05), and the ratio of CD4^+^/CD8^+^ was also raised (*P* < 0.05), compared with FMDV group (Table 2). It indicated that oral administration with POL-P3b could promote cellular immunity in mice immunized with FMD vaccine.

**Table 2:** Effect of POL-P3b on spleen lymphocyte subpopulations in mice ($\overline{\mathrm{x}}\pm \mathrm{s}$)

Group	CD4^+^ Percentage (%)	CD8^+^ Percentage (%)	CD4^+^/CD8^+^
Control	24.15 ± 0.73	13.65 ± 0.42	1.76 ± 0.04
FMDV	26.97 ± 0.38*	15.15 ± 0.71	1.78 ± 0.06
FMDV+POL-P3b (L)	26.82 ± 0.31*	15.63 ± 0.54	1.72 ± 0.03
FMDV+POL-P3b (M)	28.75 ± 0.42*	15.94 ± 0.62	1.80 ± 0.06*^∆^
FMDV+POL-P3b (H)	30.53 ± 0.61**^∆^	16.41 ± 0.25*^∆^	1.86 ± 0.05**^∆^

Compared with control, *P < 0.05,**P < 0.01; Compared with FMDV, ^∆^*P* < 0.05. Data are presented as mean ± SD (*n* = 10)

### Effect of POL-P3b on thymus and spleen index in mice immunized with O-type FMD inactivated vaccine

The effects of POL-P3b on thymus and spleen index were showed in Table 3. Compared with the FMDV group, pre-administration of oral POL-P3b at the high concentration (H) significantly increased the thymus and spleen indices (*P* < 0.05), indicating that POL-P3b could regulate immune function by influencing the weight of the immune organ.

**Table 3:** Effect of POL-P3b on thymus and spleen index in mice ($\overline{\mathrm{x}}\pm \mathrm{s}$)

Group	Thymus index	Spleen index
Control	2.53 ± 0.41	3.52 ± 0.36
FMDV	2.57 ± 0.54	3.64 ± 0.19
FMDV+POL-P3b (L)	2.54 ± 0.24	3.65 ± 0.40
FMDV+POL-P3b (M)	2.61 ± 0.32	3.70 ± 0.45
FMDV+POL-P3b (H)	2.97 ± 0.33*^∆^	3.93 ± 0.31*^∆^

Compared with control, *P < 0.05; Compared with FMDV, ^∆^*P* < 0.05. Data are presented as mean ± SD (*n* = 10)

### Effects of POL-P3b on cytokines in mice immunized with O-type FMD inactivated vaccine

**Fig. 3:**
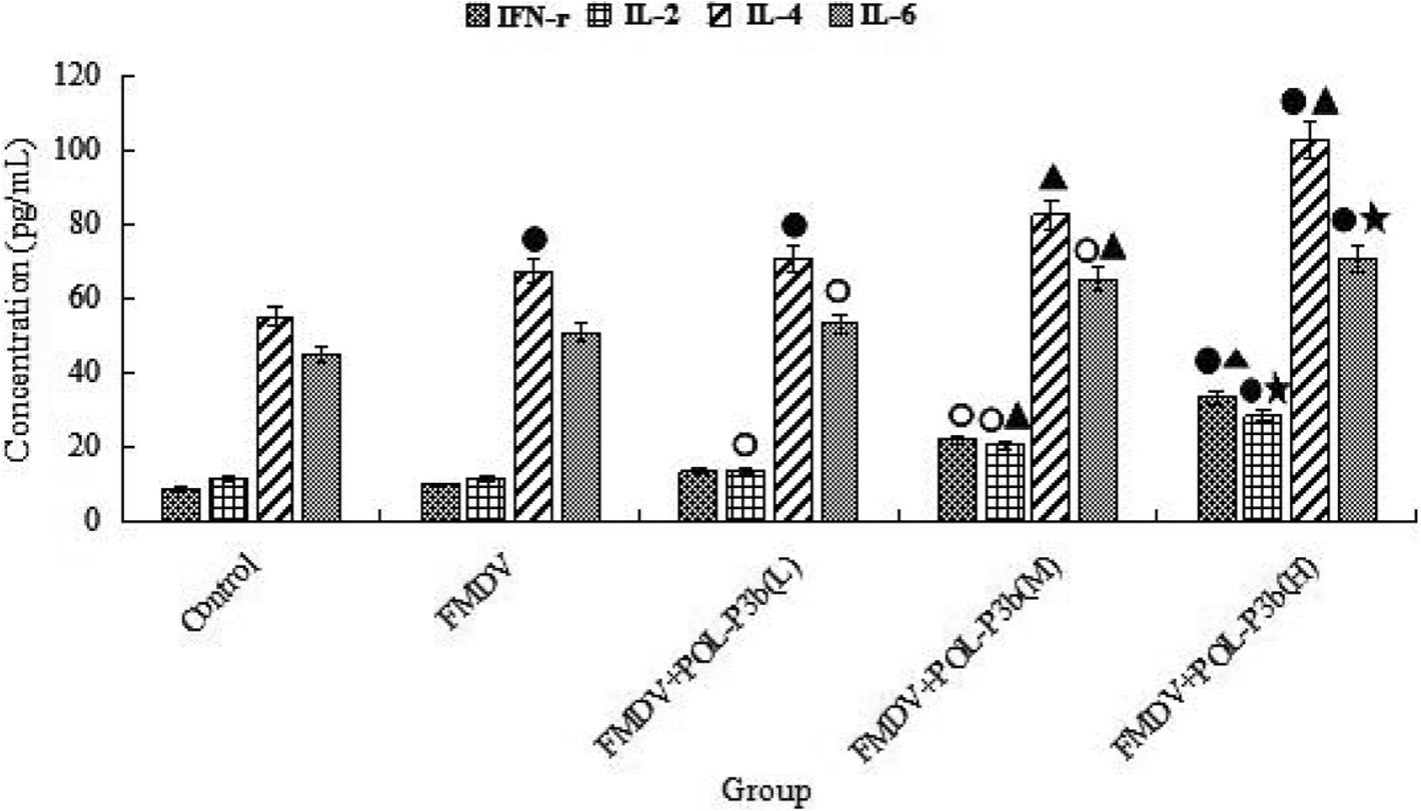
Effects of POL-P3b on cytokines in mice immunized with O type FMD inactivated vaccine. The concentrations of IFN-γ, IL-2, IL-4 and IL-6 were measured using commercially available ELISA-kits according to the manufacturer’s protocol. Data were expressed as mean ± SD (*n* = 10). ^○^*P* < 0.05, ^●^*P* < 0.01, compared with control; ^▲^*P* < 0.05, ^★^*P* < 0.01, compared with FMDV. The detection limits of these assays were 2 pg/mL (IL-2, IL-4 and IL-6) and 7 pg/mL (IFN-γ), respectively

To study if POL-P3b affected the production of cytokines in mice, the levels of various cytokines (IFN-γ, IL-2, IL-6 and IL-4) in serum were determined using ELISA. Oral administration of medium- and high-dose POL-P3b significantly increased the levels of IFN-γ, IL-2, IL-6 and IL-4 in mice immunized with FMD vaccines compared to mice in FMDV group in a dose-dependent manner (Fig. 3), suggesting that dietary supplementation of POL-P3b could promote the expression of cytokines such as IFN-γ, IL-2, IL-6 and IL-4 in mice immunized with FMD vaccines.

### Effect of POL-P3b on Ca^2+^ of lymphocyte in mice immunized with O-type FMD inactivated vaccine

**Fig. 4:**
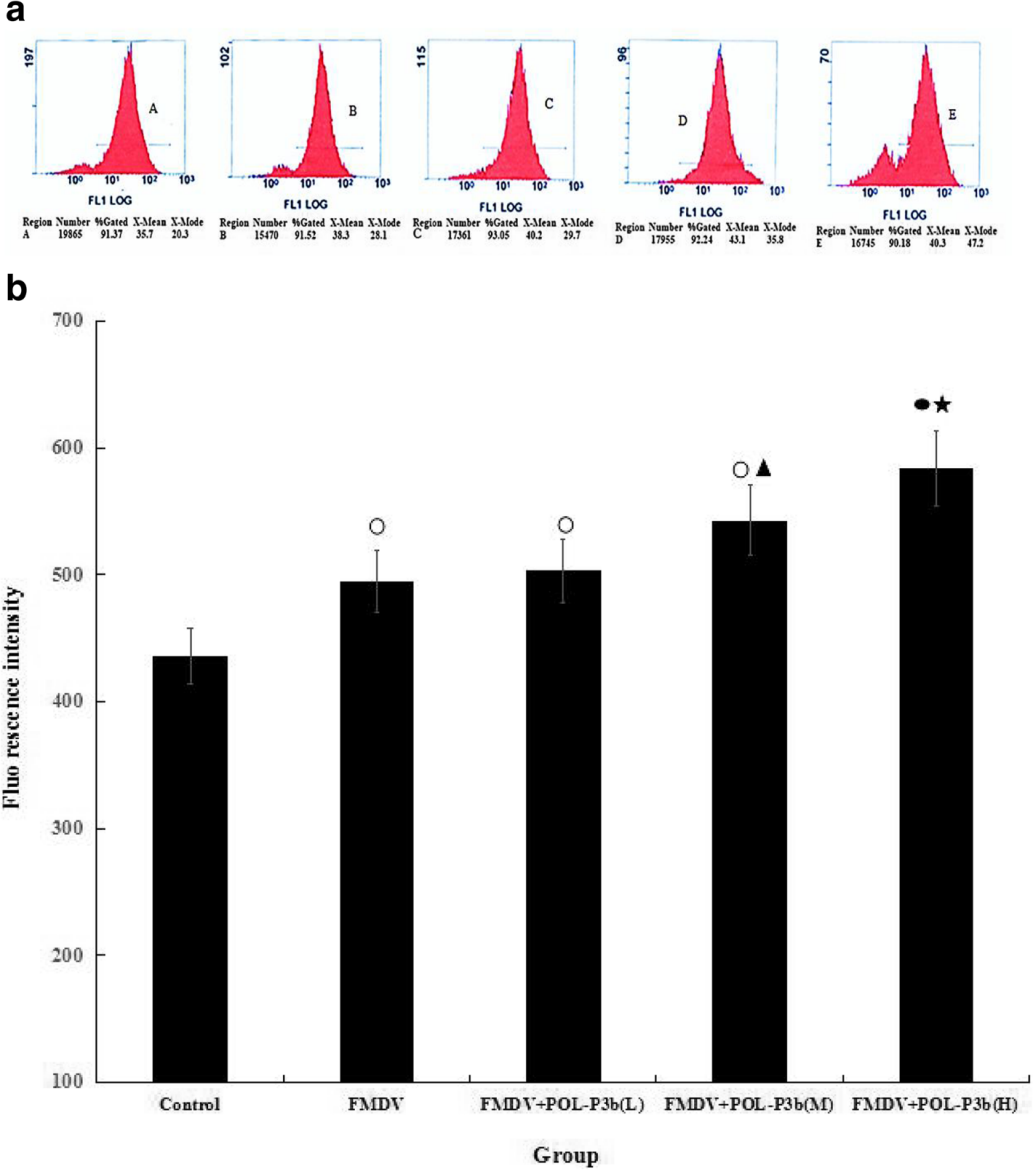
Effects of POL-P3b on Ca^2+^ of lymphocyte in mice immunized with O type FMD inactivated vaccine. **a** Lymphocyte Ca^2+^ was analyzed using flow cytometry instrument. Data were also collected in FSC (forward scatter) and SSC (side scatter) and a total of 10,000 events were collected for each sample. A Control group; B FMDV group; C FMDV+ POL-P3b (L) group; D FMDV+ POL-P3b (M) group; E FMDV+ POL-P3b (H) group **b** The level of lymphocyte Ca^2+^ was indicated with fluorescence intensity (I). The computation formula is as follows: I = Log (x-mode) × 340. Data were expressed as mean ± S.D. (*n* = 10). ^**○**^*P* < 0.05, ^●^*P* < 0.01, compared with control; ^▲^*P* < 0.05, ^★^*P* < 0.01, compared with FMDV

Calcium is a messenger molecule, that has an important effect on lymphocyte function. Intracellular Ca^2+^ in lymphocytes was measured with Fluo-3-AM using flow cytometry. It is found that FMDV immunization significantly increased content of Ca^2+^ in lymphocytes of mice relative to the control group. Furthermore, medium- and high-dose POL-P3b significantly increased the content of Ca^2+^ in lymphocyte of mice immunized with FMD vaccines compared to mice in the FMDV group (*P* < 0.05, *P* < 0.01) (Fig. 4a, b).

### Effects of POL-P3b on co-stimulatory molecules and MHC-II of intestinal DC

**Fig. 5:**
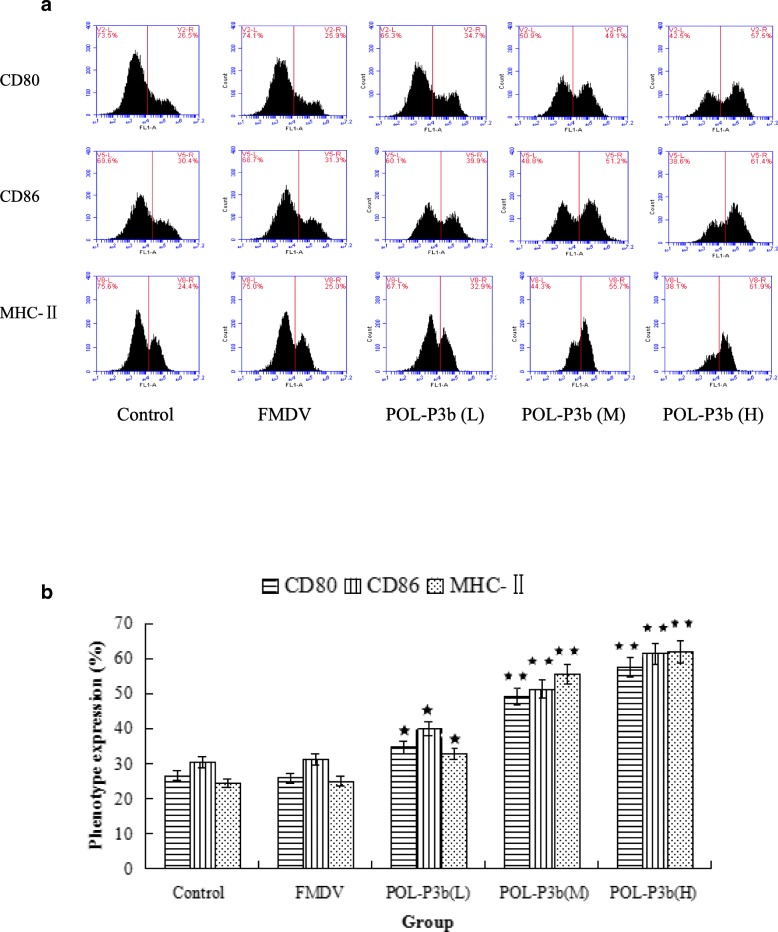
Proportion of positive cells of phenotype expression of intestinal DCs in the immunized mice treated with POL-P3b **a**. Statistical bar graph of the protein expression **b**. The quantitated values represent the mean ± S.D.Compared with FMDV, ^*^P < 0.05;^**^P < 0.01

DC maturation is characterized by the expression of co-stimulatory molecules and MHC-II molecules. Mature DC is critical for stimulating immune response. Therefore, we evaluated the capacity of POL-P3b to the maturation of DC in mice after immunization. As shown in Fig. 5a, b, oral administration with POL-P3b for mice at different concentrations increased the content of co-stimulatory molecules (CD80 and CD86) and MHC-II, and the expression in high dosed group was more significant. Our results demonstrated that POL-P3b could promote the intestinal DC maturation.

### Effect of POL-P3b on intestinal mucosa SIgA in mice immunized with O-type FMD inactivated vaccine

**Fig. 6:**
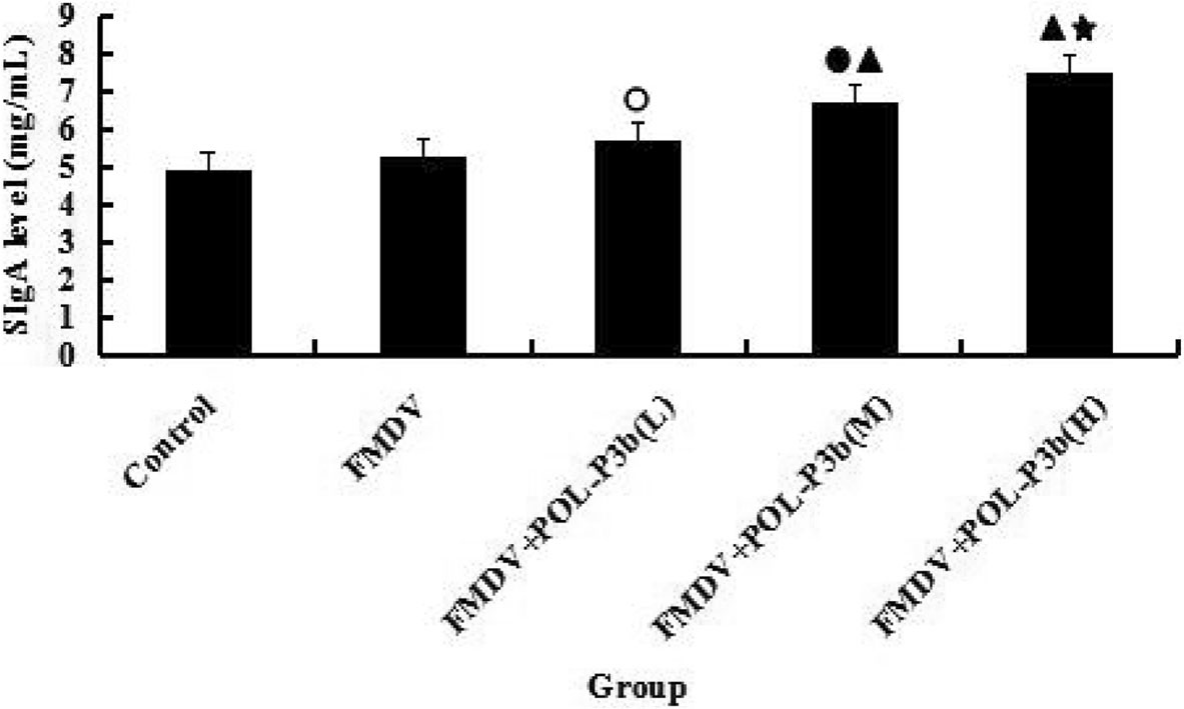
Effects of POL-P3b on intestinal mucosa SIgA in mice immunized with O type FMD inactivated vaccine. ^**○**^*P* < 0.05, ^●^*P* < 0.01, compared with control; ^▲^*P* < 0.05, ^★^*P* < 0.01, compared with FMDV

SIgA is an important effect factor of the intestinal immune barrier. To further investigate how oral administration of POL-P3b enhanced the immune response in mice, SIgA secretion in intestinal mucosa was analyzed. FMDV immunization alone did not induce secretion of SIgA in the intestine. However, treatment of medium- and high-dose POL-P3b significantly increased intestinal SIgA levels in mice immunized with the FMD vaccines (Fig. 6). The data suggested that pre-administration of oral POL-P3b was able to enhance the immune response induced by FMDV and the mechanism was likely to promote SIgA secretion in intestinal mucosa.

### Maximum tolerance daily test of POL-P3b

**Fig. 7:**
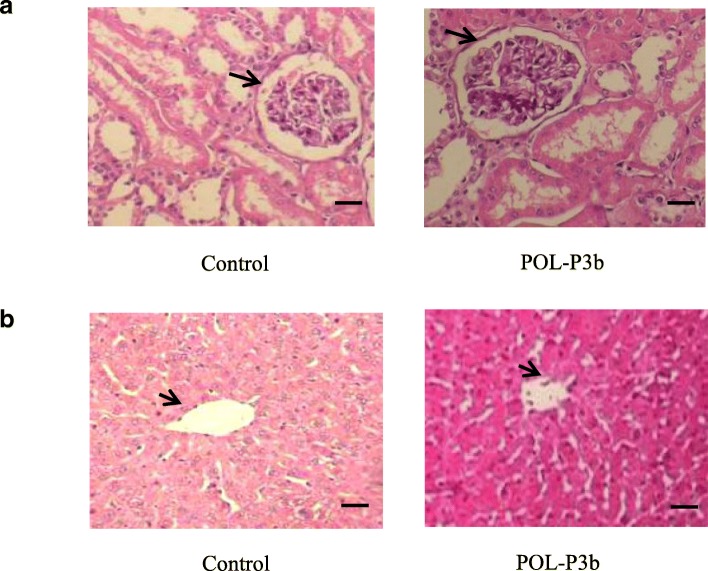
Histology of hematoxylin- and eosin-stained sections of kidney and liver in mice treated by oral administration with POL-P3b (40 × 10). **a** kidney and **b** liver. Compared to mice in control group, mice treated with POL-P3b had normal kidney structure, and renal tubular and glomerular structures were clear. Mice treated with POL-P3b had normal liver structure, with clear hepatic vein in the middle and without visible degeneration, necrosis and inflammatory cells infiltration. Glomerular was indicated with the arrow; Central vein was indicated with the arrow. Scale bars, 100 μm

Maximum tolerance daily of POL-P3b was tested by treating mice with the greatest dosage of POL-P3b for 7 days. It was found that POL-P3b was not lethal to mice and did not cause any abnormal behavior. Histological observation of kidney tissue showed that, compared to mice in control group, mice treated with POL-P3b had normal kidney structure, and renal tubular and glomerular structures were clear. There was no atrophy or compensatory hypertrophy in renal tubules (Fig. 7a). There were no statistically significant differences in the number of glomeruli, glomerular diameter and renal capsule width between the two groups (Table 4). Similarly, mice treated with POL-P3b had normal liver structure, with clear hepatic vein in the middle and without visible degeneration, necrosis and inflammatory cells infiltration (Fig. 7b). Nucleoplasmic ratio (nuclear area/cell area) of hepatocytes is one of the main characteristics for hepatocytes, and it is a common indicator in liver experiments [14]. The results showed that there was no significant difference in the nucleoplasm ratio between POL-P3b group and control group (Table 5). The above results indicated that POL-P3b was safe and nontoxic.

**Table 4:** Effect of POL-P3b on the index of glomerular tissue structure ($\overline{\mathrm{x}}\pm \mathrm{s}$, *n* = 10)

Group	Glomerular (number/view)	Glomerular diameter (μm)	Width of renal capsule (μm)
Control	17.75 ± 1.03	625.32 ± 34.21	25.33 ± 3.02
POL-P3b	16.98 ± 1.21	631.57 ± 43.24	26.05 ± 2.91

**Table 5:** Effect of POL-P3b on the index of hepatocyte ($\overline{\mathrm{x}}\pm \mathrm{s}$, *n* = 10)

Group	Area of cell (μm^2^)	Area of nucleus (μm^2^)	Area of cytoplasm (μm^2^)	Nucleoplasm ratio
Control	1453.25 ± 327.38	217.26 ± 49.03	1253.99 ± 434.52	0.1493 ± 0.017
POL-P3b	1471.47 ± 403.21	221.34 ± 57.15	1250.13 ± 421.23	0.1502 ± 0.015

## Discussion

Development of a high efficiency and low toxicity immunostimulator is an effective strategy to enhance immune responses to inactivated vaccines [15]. In general, the traditional inactivated vaccine is thought to induce a strong humoral immune response but a weak cell-mediated immune response [16]. Ideally, a potent immunostimulator should be able to enhance both humoral and cell-mediated immune responses to vaccines. At the same time, it has to be relatively safe with less toxic side effects [17]. It is reported that polysaccharides could be used as immune stimulants to affect cytokine levels and promote antibody production [18, 19]. In addition, mutual recognition between polysaccharides and complement proteins in the blood could enhance the ability of infectious microbes to resist phagocytosis [20].

Humoral immunity mediated by B lymphocytes plays an essential role in FMDV immunity. The protective immune response induced by FMD vaccines was closely related to a broad range of antibodies, such as IgG1, IgG2a, IgG2b and IgG3 [21, 22]. The production of various antibody subclasses is driven primarily by T lymphocytes and their secretion of cytokines [23]. In order to protect against intracellular pathogens, Th1 immune response have to be effectively activated, including the production of cytokines such as IFN-γ and IL-2 and the elevated levels of IgG2a, IgG2b and IgG3. In addition, Th2 immune responses, such as the secretion of IL-4, IL-6 and IgG1, could effectively remove pathogens in body fluids. Our results showed that oral administration of POL-P3b could increase the levels of FMDV-specific antibodies. Meanwhile, Th1/Th2 cytokines (IL-2, IFN-γ/IL-4, IL-6) in mice treated with oral POL-P3b were significantly higher than those in the FMDV group. These results suggested that POL-P3b could simultaneously enhance Th1- and Th2-type immune responses, which was similar to other studies [24].

Lymphocyte mediated cellular immunity plays a key role in resistance to intracellular infection. Moreover, a growing number of studies have found that cellular immunity provide effective protection against FMD [25, 26]. The ability to induce cellular immune response is detected by lymphocyte proliferation in vitro. It is generally known that Con A mainly stimulates T cell proliferation, and LPS mainly stimulates B cell proliferation. Our results suggested that immunization with inactivated FMDV alone could only stimulate humoral immunity in mice. However, POL-P3b administration could simultaneously stimulate humoral immunity and cellular immunity by increasing lymphocyte proliferation and the ratio of CD4^+^/CD8^+^ in immune mice.

Calcium ion is an important second messenger that is essential for various lymphocyte functions, such as regulation of the division of lymphocytes, promotion of IL-2 secretion, and mediation of phagocytosis by macrophages [27]. It is reported that polysaccharides could change intracellular Ca^2+^ levels in lymphocytes [28, 29]. Our results demonstrate that POL-P3b increased Ca^2+^ concentration in lymphocytes of mice immunized with FMD vaccines, indicating that POL-P3b could enhance lymphocyte functions in mice immunized with FMD vaccines.

Natural and efficient immunologic stimulants should be relatively safe and non-toxic. Our results showed that POL-P3b did not affect the tissues of liver and kidney or general behavior of the mice in the whole experiment, indicating that POL-P3b administered by mouth was safe. In addition, immune organ index is one of the important indicators reflecting the immune status. If an immunologic stimulant is used improperly, it might cause severe stress response in the animal, resulting in atrophy of immune organs. In this work, we found that POL-P3b significantly improved the spleen and thymus indices in mice, showing that POL-P3b could promote the development of immune organs.

The intestine can not only digest and absorb nutrients, but also plays an important role in the immune system. As the largest lymphoepithelial organ, the intestine contains various types of antibody-producing cells [30]. A common mucosal immune system exists in the body. Innate immune response is activated by common mucosal immune system when the local mucosal immune is enhanced, and then trigger a systemic immune response [31]. The current research is more focused on the intestinal mucosal immune system, which is the first barrier for intestinal infection caused by bacteria, virus and parasite [32]. Since the oral administration is the most common route for the patients to take the traditional Chinese medicine (TCM), the medicines have direct effects on the digestive tract. TCM is rich in a variety of bioactive ingredients and nutrients, which can provide energy and nutrition to maintain intestinal mucosal immunity and barrier. It is reported that the intestinal mucosal immune system is crucial to maintain the integrity of the digestive organ and plays a key role to local immune regulation [33]. Therefore, to explore the effects of POL-P3b on the intestinal mucosal immune system through oral pre-administration is of a great significance to prevent FMD infection. The experimental results showed that oral administration with POL-P3b in mice at different concentrations increased the content of co-stimulatory molecules (CD80, CD86) and MHC-II. Our results suggested that POL-P3b can promote the maturation of DC in the intestinal mucosa, thus improve the ability of the antigen presentation, and then enhance the intestinal mucosal immunity and specific immune response. These experimental data further demonstrated that the increased lymphocyte proliferative response was paralleled with the enhanced gut mucosal immunity.

Secretory immunoglobulin A (SIgA) is a major effector in the intestinal mucosal immune system that plays a vital role in innate and adaptive immunity [34]. SIgA prevents microorganisms and pathogens from adhesion to the intestinal tract, and activates the complement component 3 (C3) bypass pathway [35]. For the first time, we found that oral administration of POL-P3b could significantly increase the levels of SIgA in mice. The increase in mucosal SIgA secretions suggest that oral administration of POL-P3b might activate the intestinal mucosal immune response, and further improve immune response of the organism to FMD vaccines. However, the detailed mechanism is still unknown and needs to be further studied.

## Conclusions

In conclusion, our results have provided strong evidence that dietary POL-P3b supplementation could enhance the immune response, including humoral and cellular responses, to FMD vaccines, and the mechanism was likely to enhance the intestinal mucosal immunity and promote SIgA secretion in intestinal mucosa.

## Methods

### Materials

POL-P3b was purified from Purslane by DEAE cellulose and Sephadex G-200 column chromatography.7 Cell Counting Kit-8 (CCK8) was purchased from Sigma-Aldrich. The ELISA kits of IL-2, IL-4, IL-6, IFN-γ and SIgA were purchased from Beyotime Biotechnology (Jiangsu, China). Type O FMD inactivated vaccine was provided by the Inner Mongolia Jinyu pharmaceutical factory (Mongolia, China). The vaccine contains an inactivated OHM/02 strain of FMD Type O virus, and the virus content before inactivation should be at least 10^7.0^ LD _50_/0.2 mL. The FMD antibody ELISA test kits were purchased from the Lanzhou Veterinary Research Institute of the Chinese Academy of Agricultural Sciences (Lanzhou, China).

### Animals and immunization

Female BALB/c mice (6–8 w, 18–22 g) were purchased from the Experimental Animal Center of Harbin Medical University. The experimental mice were housed under a suitable temperature (23 ± 3 °C) and humidity (55 ± 15%) with a 12 h light/dark cycle. The mice were allowed free access to clean water and food. A stock solution of POL-P3b was prepared by diluting 5 mg purified POL-P3b in 1 mL of 0.9% saline solution and stored at 4 °C until for use. Fifty mice were randomly divided into five groups with 10 mice in each group, including control a group (0.9% saline solution, without subsequent vaccination), a FMDV group (0.9% saline solution), low-dose group (2 μg/mL POL-P3b), medium-dose group (10 μg/mL POL-P3b), and high-dose group (50 μg/mL POL-P3b). All animals were administered POL-P3b orally in an equivalent volume of 0.2 mL/10 g body weight on a daily basis for 4 days. At 24 h after the last administration of POL-P3b, Type-O FMD vaccines (0.2 mL per mouse) was subcutaneously injected into the right groin of mice (except those of the control group). Two weeks after the initial vaccination, the mice received a booster immunization. Two weeks after the booster immunization, the mice were anesthetized using 2.5% isoflurane, and the best effort was employed to minimize the pain, and sacrificed by exsanguination. The blood and tissues (spleen, thymus and intestines) were collected for subsequent assays.

### Viral challenge

To identify the protective effects and the safety of POL-P3b, protection against FMDV in mice was performed. Twenty mice in each experimental group were intraperitoneally (i.p.) inoculated with 10^5.0^ TCID_50_/ 0.1 mL infectious FMD Type O virus at 21 days post vaccination (dpv). The death of the animals was counted every day after inoculation. The survival rate of each group was calculated according to the number of survival mice after recovery.

### Determination of IgG and isotype levels

To determine the effect of POL-P3b on antibody production, blood samples were collected from mice (10 mice in each group) 2 weeks after booster immunization, and the FMDV-specific IgG titres and isotypes in serum were detected using ELISA. Briefly, 96-well plates were coated with 50 μL of rabbit anti-FMDV serum diluted in 0.05 M carbonate-bicarbonate buffer (1:800) overnight at 4 °C. After washes with phosphate-buffered saline (PBS) containing 0.05% Tween 20 (PBST), the wells were blocked with 5% skim milk in PBS for 2 h at room temperature. After washes with PBST, 50 μL of Type O FMDV antigen (dilution: 1:8 PBS) were added into each well, followed by incubation at 4 °C for 2 h. After washes with PBST, 50 μL of serum obtained from mice (1:40000 in PBS containing 5% skim milk) were added into the wells, and the plate was incubated at 37 °C for 1 h. After washes with PBST, horseradish peroxidase-conjugated antibodies against IgG, IgG1, IgG2a, IgG2b or IgG3 (1:5000 in PBS containing 5% skim milk) were added into the wells, followed by incubation at 37 °C for 1 h. After washes with PBST, 50 μL of TMB substrate solution were added and incubated at 37 °C for 15 min, and reaction was stopped with 50 μL of 2 M H_2_SO_4_. The absorbance at 450 nm was measured using a microplate reader (PT-DR200Bs, Beijing Potenov Tech., Beijing, China).

### Splenocyte proliferation and CD4^+^/CD8^+^assay

After the mice (10 mice in each group) were sacrificed, spleens were collected to prepare lymphocyte suspension. A single cell suspension was prepared by gentle teasing of the spleen in ice-cold Hanks’ solution, followed by centrifugation (1500 rpm, 10 min). The cell pellet was resuspended in 2 mL red blood cell lysis buffer, and incubated at 37 °C for 15 min. After centrifugation, cells were washed three times with RPMI-1640 medium. Cells were re-suspended in RPMI 1640 medium and incubated at 37 °C for 2 h in a humidified 5% CO_2_ incubator in order to remove adherent cells. Cell viability was measured by the Trypan Blue dye exclusion test and was always higher than 95%. Aliquots (100 μL) of lymphocyte suspension (1 × 10^6^ cells/mL) were seeded into each well of a 96-well plate in triplicates, and incubated for 12 h. Then, 100 μL of RPMI-1640 media containing ConA and LPS were added into each well at a final concentration of 5 μg/mL (ConA) and 10 μg/mL (LPS), respectively. After 20 h of incubation, proliferation of both T cells and B cells was then determined using a Cell Counting Kit-8 kit (CCK8) (Sigma-Aldrich) according to the manufacturer’s instructions. The absorbance at 450 nm was measured using a microplate reader (Bio-Red). The lymphocyte stimulation index (SI) was calculated as follows:

SI = (OD value of cells with stimulation - OD value of blank wells)/(OD value of cells without stimulation - OD value of blank wells).

Cell suspensions (1 × 10^6^ cells/mL) were centrifuged at 1000 rpm for 5 min, and the pellets were re-suspended with Hanks solution. The antibody (CD3, CD4 and CD8) was added and incubated at room temperature in the dark for 30 min. After washing three times, the levels of antibodies were measured using flow cytometry (CytoFLEX, Beckman Coulter, USA).

### Determination of thymus and spleen index

After the mice (10 mice in each group) were sacrificed, the thymus and spleen tissues were rapidly isolated under aseptic conditions and weighed. The thymus index and spleen index were expressed as the ratio of thymus and spleen weight relative to body weight (mg/g), respectively.

### Determination of serum cytokines

To observe if POL-P3b affect cytokine production, the levels of various cytokines (IL-2, IL-4, IL-6 and IFN-γ) in serum were measured using enzyme-linked immunosorbent assay (ELISA) according to the manufacturer’s protocol (Beyotime Biotechnology, Jiangsu, China).

### Determination of Ca^2+^ content in lymphocytes

Single cell suspension of 10% chicken red blood cells was used as an internal standard. Briefly, lymphocytes were suspended in RPMI-1640 media containing 5 μM Fluo 3 (a Ca^2+^ indicator), and incubated in the dark for 30 min. Then, cells were washed three times with RPMI-1640 medium and resuspended in RPMI-1640 medium. Fluorescence intensity of lymphocytes was measured on a CytoFLEX flow cytometer (CytoFLEX, Beckman Coulter, USA).

### Expression of DC surface co-stimulatory molecules and MHC-II by flow cytometry

Mice (10 mice in each group) were sacrificed, and the small intestine and the ileum were taken out, followed by removal of the membrane and fat. After the intestinal cavity was flushed using PBS solution containing 10% fetal bovine serum (operating on the ice), the intestinal tube was cut into 2 mm^3^ tissue block with scissors. All the tissue blocks were digested with the digestive fluid I (PBS + 1 mM EDTA+ 1 mM DTT + 10% FBS) at 37 °C for 40 min. Tissue blocks was blown with a straw repeatedly and centrifuged for 5 min, then the tissue continued to be digested using digestive fluid II (PBS + 100 U/L collagenase IV + 10% FBS). The digested tissue suspension was filtered through the 100 and 500 stainless steel wire meshes. The isolated cells were counted and resuspended in RPMI1640 supplemented with 10% fetal bovine serum, 100 U/mL penicillin, 100 μg/mL streptomycin, 2 mM L-glutamine, 50 μM 2-mercaptoethanol, 20 ng/mL of rmGM-CSF and 1 ng/mL rmIL-4 at 1 × 10^6^ cells/mL. On the day 6, DCs were harvested and cells with CD11c expression were isolated using MACS according to the manufacturer’s recommended protocol, which were later seeded into multi-well plates with fresh medium. Cells were collected and stained using PE- and FITC-conjugated antibodies. Fluorescence intensities were detected by Flowcytometry.

### Determination of SIgA

After the mice (10 mice in each group) were sacrificed, the duodenum, jejunum and ileum were taken and then the intestinal tube was cut longitudinally for scraping the intestinal mucosa. All mucosal secretions were stored at − 20 °C for IgA analysis. The SIgA levels in mucosal secretions were determined using ELISA according to the manufacturer’s protocol (Beyotime Biotechnology, Jiangsu, China).

### Maximum tolerance daily studies

In the preliminary experiment, acute oral toxicity assessment of POL-P3b (7000 mg/kg body weight) did not cause mortality, abnormal clinical signs or any significant pathological changes. The results indicated that POL-P3b had little toxicity and LD50 could not be detected. Therefore, the maximum tolerance daily (MTD) is measured according to the New Drug Approval Procedure [36].

Healthy BALB/c mice (6–8 weeks, 18–22 g) of either sex (10 mice in each group), were treated with the POL-P3b solutions of the maximum concentration (20 mg/mL), and the mice were orally fed with POL-P3b solutions of 0.4 mL/10 g body weight (the maximal dose of gavage for mice) once a day for 7 days. The general behavior and neurological profiles of mice were observed, such as fur color, body weight, food intake, urine and stool, etc. For histopathological observation of kidney and liver at 7 days after administration of POL-P3b, the tissues were fixed in PBS buffer containing 10% formalin (pH 7.4) for over 24 h and then embedded in paraffin. Thin sections (5 μm in thickness) were prepared using a microtome (RM2235, Leica) and stained with hematoxylin and eosin. The sections were observed and photographed with a microscope (XSP, Teelen, Shanghai, China). Morphological changes of glomeruli and tubules were observed and the number of glomeruli was counted at 100 x field of view. The largest 10 glomerulus were selected for each slice under the 400 x field of view, and the diameter of glomerulus and the width of renal capsule were measured using Image analysis software (Image-pro Plus 6.0). Different fields of view were selected under 400 x microscope, and the area of liver cells and nucleus was measured using Image-pro Plus 6.0, then the cytoplasmic area and nucleoplasmic ratio were calculated.

### Statistical analysis

All data are presented as means ± standard deviation (SD). Statistical analysis was performed using SPSS 20.0. Statistical significance was analyzed by one way analysis of variance (ANOVA) with differences between groups using Dunnett’s post hoc tests. Differences were considered statistically significant when *P* < 0.05.

## Abbreviations

CCK8: Cell Counting Kit-8
FMD: Foot-and-mouth disease
FMDV: Foot-and-mouth disease virus
IFN-γ: Interferon-γ
POL-P3b: Purslane polysaccharide
SI: Stimulation index
SIgA: Secretory immunoglobulin A
